# Mental health consequences of COVID-19: the next global pandemic

**DOI:** 10.1590/2237-6089-2020-0081

**Published:** 2020-10-08

**Authors:** Jair de Jesus Mari, Maria A. Oquendo

**Affiliations:** 1 Departamento de Psiquiatria Escola Paulista de Medicina Universidade Federal de São Paulo São PauloSP Brazil Departamento de Psiquiatria , Escola Paulista de Medicina , Universidade Federal de São Paulo (UNIFESP), São Paulo , SP , Brazil .; 2 Standing Committee on Science, Education and Publication World Psychiatric Association Geneva Switzerland Standing Committee on Science, Education and Publication , World Psychiatric Association , Geneva , Switzerland .; 3 Perelman School of Medicine University of Pennsylvania PhiladelphiaPA USA Perelman School of Medicine , University of Pennsylvania , Philadelphia , PA , USA .; 4 American College of Neuropsychopharmacology BrentwoodTN USA American College of Neuropsychopharmacology , Brentwood , TN , USA .; 5 American Foundation for Suicide Prevention New YorkNY USA American Foundation for Suicide Prevention , New York , NY , USA .

As the population is exposed to traumatic scenes due to the coronavirus disease 2019 (COVID-19), either in real life or through media from all over the planet, the emergence of mental disorders in vulnerable individuals is guaranteed. The most common disorders seen after a catastrophe are major depression, post-traumatic stress disorder, and anxiety disorders; increases in alcohol and drug use are also observed. To call this stress catastrophic is not hyperbole. It is catastrophic because the impact of COVID-19 on mental health will be due to at least five different effects of the pandemic, each of which is expected to independently have profound effects on mental health. This suggests that mental health sequelae will be greater than those seen after other disasters. ^1^

The first impact of COVID-19 on mental health is the sudden – and in some regions, unexpected – arrival of the virus, which left cities deserted, bringing fear and triggering acute stress reactions. The fear of being contaminated or of contaminating others is no different from that seen after traumatic situations such as an earthquake or other disaster.

The second effect impacting mental health is the need for quarantine. While quarantine is necessary for fighting the pandemic, the sudden change in routine and the confinement can lead to feelings of helplessness, boredom, anxiety, anguish, irritability and anger at the loss of freedom. These reactions can be simply a situational adjustment to the new reality and not necessarily pathological. After all, being depressed and anxious is a normal reaction to the existing insecurity. Nonetheless, the mental health effects of quarantine themselves are remarkably similar to those of traumatic events. ^2^

The third effect impacting mental health relates to the alarmingly numerous deaths resulting from COVID-19 – overwhelming hospitals, mortuaries, and funeral homes. Without the usual farewell rituals, such as spending time with the person as they are dying or having funerals, cases of complicated grief with depression and risk of suicide may increase. ^3^

Yet another COVID-19 effect on mental health relates to the individual perceptions of those admitted to intensive care units, who will experience searing, terrifying phenomena, with some of them developing future episodes of major depression, post-traumatic stress disorder, and other psychiatric conditions.

Finally, the economic losses, unemployment, food insecurity, and increased social inequality are all generating acute stress likely to morph into chronic stress for a large swath of the population, also increasing risk for mental disorders. At the same time, the impact of this new wave of mental disorders on the economy is not to be underestimated. ^4^

The population most severely exposed to stress during COVID-19 are the health professionals on the frontlines. They are subject to significant physical and emotional demands, often with insufficient assistance or personal protective equipment to guarantee safety. Add to this the daily suffering witnessed, and the difficult ethical decisions to be made, and the situation for those on the frontlines is daunting, to say the least. ^5^

To understand mental health symptoms, the National Institute of Mental Health (NIMH) website is a good resource. ^6^ The best indicator of emotional health is maintenance of functionality, despite a routine that is new and unstructured for many. If the anguish or depression is uncontrollable or impacts other aspects of life, such as family or professional function, it is time to seek professional help.

The World Psychiatric Association (WPA) established an Emergency Response Committee to ensure a centralized, coordinated response to COVID-19. Also, WPA is creating a library of resources, including instruction manuals and materials to support healthcare staff working with COVID-19 patients that will be freely available to the public in simple and immediate ways (several materials in different languages are already available). ^7^

Because these phenomena are occurring at the global level, humanity has never needed mental health professionals as much as it does now. The predictable, multiple psychological issues deriving from COVID-19 will demand a much more organized response than is currently possible due to the lack of psychiatric care in many parts of the globe, especially in low- and middle-income countries. ^8^ Mental health care at the global level will have to reinvent itself, and the crisis precipitated by the pandemic presents an opportunity to make mental health care as available as possible.
